# Molecular Insights into High-Pathogenicity RNA Viruses

**DOI:** 10.3390/v18080912

**Published:** 2026-08-19

**Authors:** Hana Krnjić, Adna Hrapović, Aiša Galijatović, Ajla Tipura, Maida Hajdarpašić, Selma Kozarić, Adna Berilo, Naida Odobašić, Altijana Hromić-Jahjefendić, Jasmin Šutković

**Affiliations:** Genetics and Bioengineering, Faculty of Engineering and Natural Sciences, International University of Sarajevo, Ilidža, 71000 Sarajevo, Bosnia and Herzegovina; 220301107@student.ius.edu.ba (H.K.); aisatrebo@hotmail.com (A.G.); ajlatipura1@gmail.com (A.T.); 250301014@student.ius.edu.ba (A.B.); 250301013@student.ius.edu.ba (N.O.)

**Keywords:** RNA viruses, antiviral targets, SARS-CoV-2, Ebola virus, Influenza A virus, biomolecular condensates, liquid–liquid phase separation, host–virus interactions

## Abstract

Highly pathogenic RNA viruses, such as Ebola, SARS-CoV-2, and influenza, cause severe disease in humans. High mutation rates, which enable RNA viruses to evade immunity and escape antivirals, and their ability to spread from animals to humans and cause pandemics and outbreaks, make RNA viruses significant threats to public health. Diseases caused by Ebola, SARS-CoV-2, and influenza are prevented and treated with only a limited number of approved antiviral drugs, the effectiveness of which is limited by mutations in the viral targets. It is crucial to understand the structural determinants, molecular mechanisms, and host interactions of pathogenic RNA viruses to develop effective antiviral strategies. In this review, we discuss selected RNA viruses, focusing on the structure of their RNA polymerases and interactions with host factors during the different stages of the viral lifecycle, as well as the traditional antivirals targeting these structures and pathways. Furthermore, emerging concepts such as liquid–liquid phase separation and biomolecular condensates, and novel promising antiviral strategies are discussed. Understanding shared and distinct structures, molecular mechanisms, and host interactions across highly pathogenic RNA viruses enables the discovery of new and more effective antiviral strategies, ultimately improving clinical outcomes against evolving RNA viruses.

## 1. Introduction

RNA viruses are characterized by having an RNA genome, excluding the DNA molecule from the processes of replication and gene expression. Within the classification system known as the Baltimore classes, RNA viruses fall under three categories, each distinguished by the nature of the genome, specifically, the type of nucleic acid present in the genome. These Baltimore classes correspond to distinct strategies for replication and gene expression: positive-sense (+) RNA viruses, double-stranded RNA viruses, and negative-sense (−) RNA viruses. Positive-sense (+) RNA viruses employ a straightforward replication and expression mechanism where the same RNA molecule serves as both the genome and mRNA [1]. The largest and most diverse class of viruses that infect eukaryotic organisms is the positive-sense single-stranded RNA viruses. The coding-sense RNA segments that make up the genomes of these viruses serve as messenger RNAs as soon as they enter the cytoplasm of infected cells. In contrast, negative-sense (−) RNA viruses carry genomes that are complementary to mRNA and therefore cannot be translated directly upon entry into the host cell. Instead, they must package a viral RNA-dependent RNA polymerase (RdRp) within the virion to synthesize positive-sense mRNAs and replication intermediates immediately after infection [1]. RNA viruses replicate their genomes using RNA-dependent RNA polymerase (RdRp) and exhibit either a spherical or a rod-shaped (helical) structure. While replicating, RNA viruses engage in synthesizing at least three distinct types of RNA, including a genome, a copy of the genome, and messenger RNA (mRNA) [1]. They are known to adjust fast to new environments and go through evolutionary changes, which can result in higher mutation rates and the formation of new viral strains [2]. RNA viruses come in 180 recognized species, and two new species are found annually on average. RNA viruses are commonly spread between humans and other hosts, primarily mammals and occasionally birds, both epidemiologically and evolutionarily [3].

Various diseases, including Ebola, influenza, and COVID-19, are known to be caused by RNA viruses. The symptoms can range from mild, such as a cold, fever, and sore throat, to more severe conditions like respiratory diseases, liver failure, or even death, depending on the specific virus involved [2]. SARS-CoV-2, the virus causing COVID-19, influenza, and Ebola virus, the cause of Ebola virus disease (EVD), are single-stranded RNA viruses. Influenza and Ebola virus have a negative-sense (−) RNA genome, while SARS-CoV-2 has a positive-sense RNA genome. The virions of these three viruses are enveloped, with a distinct morphology characterizing each—long, thread-like Ebola, spherical SARS-CoV-2, and pleomorphic (variable) influenza virions [4,5,6].

A special concern with diseases caused by pathogenic RNA viruses is the potential for outbreaks and pandemics, as they can often be spread between animals and humans, as well as readily between humans [3]. Immunization is a highly effective method for preventing certain infectious diseases caused by RNA viruses, as evidenced by the success of vaccines against poliovirus, SARS-CoV-2, measles virus, influenza virus, etc. Still, prevention may fail, and the availability of antiviral strategies is critical [7]. At present, the dominant approach in combating RNA viruses involves the use of antiviral drugs: special agents designed to inhibit the multiplication and spread of viruses in the host. However, given that RNA viruses possess a compact genome and exhibit a high mutation rate, conventional antiviral drugs face considerable challenges in effectively treating them alone. Consequently, the demand for additional approaches in both treatment and prevention has emerged. Progress in understanding structural determinants, molecular mechanisms of recognition, entry, RNA synthesis, and immune evasion, as well as the host interaction of RNA viruses, reveals new potential targets that can be exploited for antiviral development. This review aims to provide an assessment of structures and molecular mechanisms of selected RNA viruses currently exploited as antiviral strategies and highlight emerging concepts and novel strategies in combating them.

## 2. RNA Polymerases and Determinants of Genome Evolution

RNA viruses replicate with RNA-dependent RNA polymerase (RdRp). Though a crucial enzyme needed for the replication and transcription of RNA viruses, the RdRp genes are highly divergent, leading to distinct structural organization of replication complexes in different viruses. Still, the core unit utilized for RNA synthesis is conserved across viruses and consists of a right-hand-like structure, with palm, fingers, and thumb domains [2].

**Figure 1:**
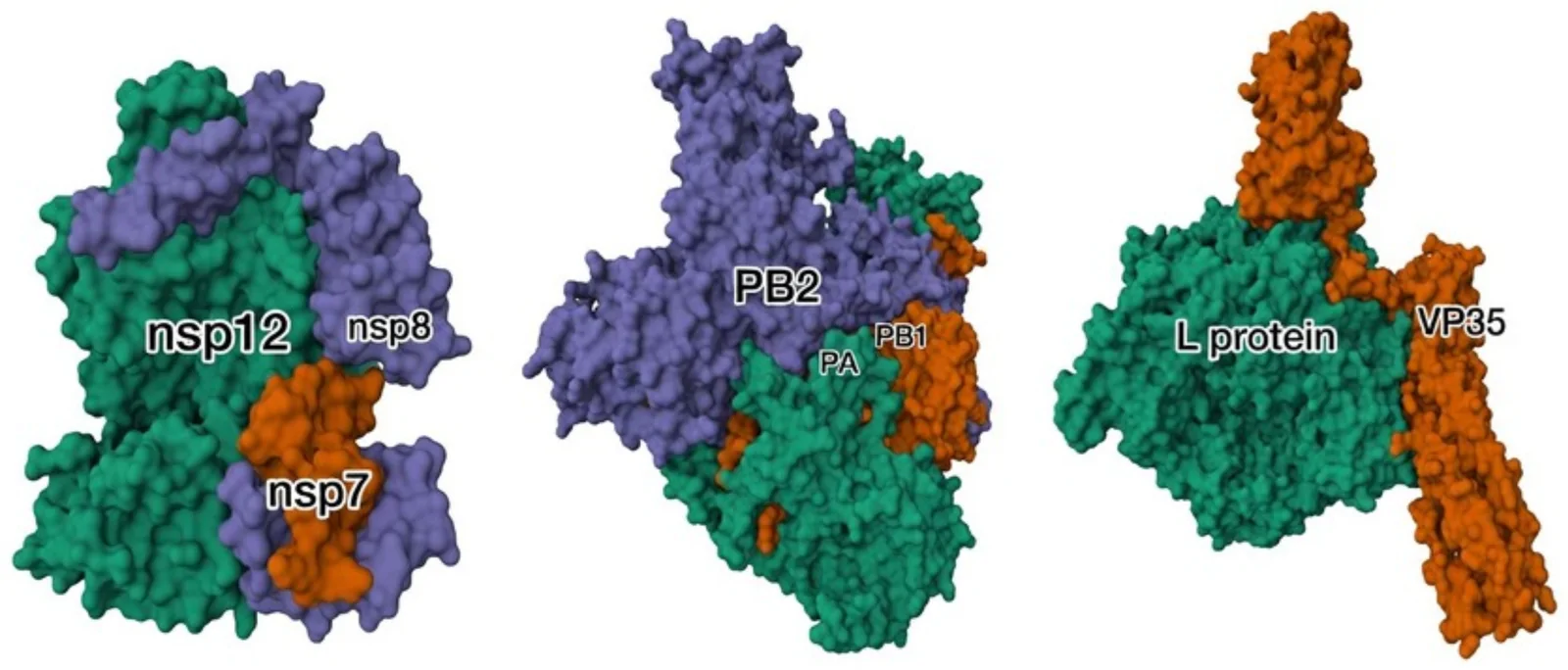
Structures of the SARS-CoV-2, influenza, and Ebola virus RNA-dependent RNA polymerases (RdRps). Although SARS-CoV-2, influenza virus, and Ebola virus all rely on RNA-dependent RNA polymerases for genome replication and transcription, their polymerases differ substantially in architecture and functional organization. The SARS-CoV-2 structure shown (**left**) represents the core RdRP complex composed of nsp12 bound to its cofactors nsp7 and nsp8, which together catalyze RNA synthesis (green color = nsp12, red color = nsp7 and purple color = nsp8). Additional non-structural proteins involved in replication and RNA processing, including the proofreading exonuclease nsp14 and proteins participating in RNA capping, are components of the broader replication-transcription complex but are not depicted in this structure. In contrast, the Ebola virus RdRp consists of the multifunctional L protein, which performs RNA synthesis, capping, and methylation in association with its cofactor VP35 (**right**). The influenza virus polymerase is a heterotrimeric complex comprising PB1 (red color), which catalyzes RNA synthesis, PB2 (purple color), which mediates host cap recognition, and PA (green color), which provides endonuclease activity required for cap snatching (**middle**). Despite these architectural differences, the catalytic cores of all three polymerases retain the conserved “right-hand” fold characteristic of many RNA- and DNA-dependent polymerases. PDB IDs: 6M71 (SARS-CoV-2 core RdRP), 8JSL (influenza virus polymerase—green color), and 4WSB (Ebola virus L–VP35 complex—red color), shown from left to right [16,17,18,19].

RNA in SARS-CoV-2 is synthesized by the replication–transcription complex (RTC). The RTC is formed by the association of multiple non-structural proteins (nsp). The SARS-CoV-2 RdRp active site is located near the C-terminus of nsp12 and, similar to other viral RNA polymerases, has a “right-hand” structure with thumb, fingers, and palm subunits. Near the N-terminus of nsp12, connected by the interface domain, is the nidovirus RdRp-associated nucleotidyl transferase (NiRAN) domain, the complete mechanistic function of which is yet to be fully determined. To form a functional RTC, cofactors nsp7 and two nsp8 units bind to nsp12, constructing the core RdRp complex (Figure 1). Additionally, nsp14, nsp16, nsp9, and nsp10 are thought to associate with the core complex to form the final RTC [8,9]. The supporting nsp proteins (nsp9, nsp14, nsp16), together with NiRAN, mediate the RNA capping [10]. The nsp14 additionally has a proofreading activity, a mechanism not observed in other RNA viruses, likely to compensate for the mutational burden of SARS-CoV-2’s large genome [11]. During SARS-CoV-2 RNA replication, the cleft of the active site within nsp12 (specifically, the area between the fingers and thumb domains) binds the first RNA turn, and the two nsp8 units positioned on both sides of the cleft hold the second turn. The investigation into the replicating structure of SARS-CoV-2 RdRp revealed that the conformation of the RdRp is not drastically altered following RNA binding. However, it revealed a “sliding pole” feature, formed by the alpha-helical extensions of the nsp8 unit, which interacts with the exiting RNA duplex. As mentioned, the SARS-CoV-2 genome is larger than that of other RNA viruses, and this “sliding pole” feature is hypothesized to be the reason behind the RdRp processivity [12].

Given the structure of influenza RNA during RNA synthesis (segmented vRNP templates), the site of its replication, and the replication/transcription mechanism, its RdRp has distinct features compared to SARS-CoV-2 RTC. The RdRp of the influenza virus is a heterotrimeric protein composed of PA, PB1, and PB2 subunits (Figure 1). Similar to other RdRps, the catalytic subunit of influenza’s RdRp–PB1 has a right-hand-like architecture with thumb, fingers, and palm domains. The N- and C-terminus extensions connect the PB1 subunit to the PA and PB2 subunits, respectively. Influenza virus replicates in the host’s nucleus rather than in the cytoplasm; thus, its RdRp must be imported into the nucleus. Because of this, the PB1 subunit has a nuclear localization signal (NLS) in its structure, which enables the transport of this viral protein to the nucleus. The RNA binds to the PB1 subunit’s finger domain, which is fitted into the loop structure of the PA subunit, after which the new molecule is synthesized in the PB1’s catalytic domain. The complex is stabilized through the interaction between the C-terminal extension of the PB1 subunit and the 3’ end of the RNA molecule. The PA has an N-terminal endonuclease domain, while the PB2 subunit contains a cap-binding domain, both of which are characteristic of influenza’s replication mechanism [4,13].

The Ebola virus RdRp is known as the RNA-dependent RNA polymerase L or L protein, and is a single, multifunctional protein involved in RNA replication, transcription, and modification. The L protein consists of three domains, namely the RdRp domain, polyribonucleotidyl transferase (PRNTase), and methyltransferase (MTase) domain. Once again, palm, fingers, and thumb domains are present within the RdRp, and the catalytic site is located on the palm, between the fingers and thumb subunits. The active site is surrounded by four channels, serving as sites for template entrance and exit, transcript exit, and NTP access. The capping subunit (PRNTase) wraps around the transcript exit channel. At its N-terminus, the L protein interacts with the viral protein 35 (VP35), an essential cofactor of Ebola replication, which stabilizes the complex and facilitates the interaction with nucleoproteins (Figure 1) [14,15].

Aside from RNA synthesis, RdRp plays an important role in viral evolution. RNA viruses have two distinct properties that are significantly correlated: a tiny genome and a high mutation rate [3]. One mutation per genome occurs during each cycle of replication for RNA viruses that reproduce via RdRp. This suggests that mutations have played a crucial role in the evolution of RNA viruses [2]. A general trend of an increase in the RNA virus genome length as its mutation rate rises is observed. One source of variation in RNA viruses is through base misincorporation by RdRp, which leads to the rapid evolution of RNA viruses. Although a high mutation rate is beneficial for viruses, a balance between diversity and evolution, and the mutational burden and genomic instability, is required [20]. The high mutation rate of most RNA viruses can be explained by the low fidelity of RdRp. SARS-CoV-2 is a major exception, which, despite having a large genome, preserves high-fidelity replication. This can be explained by the presence of a proofreading mechanism through nsp14 [11].

In addition to RdRp replication error mutations, mutations in RNA viruses can arise from modifications by the host enzymes, such as RNA-specific adenosine deaminase (ADAR) and polyprotein editing complex B (APOBEC). ADAR proteins break down adenosine to inosine, where A transforms to G, and hence directly interact with sections of double-stranded RNA [21]. ADAR protein-induced mutations have been detected in several viruses, including SARS-CoV-2 and the influenza virus. Moreover, the Ebola virus (EBOV) could be a potential target of ADAR family proteins. The prevalence of mutations A to G (U to C in the EBOV sense chain) was demonstrated, and the ADAR modifications in the untranslated regions of Ebola mRNA were shown to affect translation [22,23]. Furthermore, it is suggested that these mutations in the Ebola glycoprotein might offer it greater diversity [23]. Data on ADAR activity and its effect on the evolution of viruses are still being collected, but overall, the evidence points to ADAR as a highly likely source of RNA variety [20].

APOBECs are antiviral enzymes facilitating a C-to-U substitution through cytosine deamination [24]. The aim of the mechanism is to inhibit viral protein synthesis through nonsense and missense mutations; however, the viral RNA modification by APOBECs also leads to viral evolution. APOBEC has been shown to modify the RNA of SARS-CoV-2, accounting for a high amount of C-to-U substitution and subsequently providing a source of selection and evolution [24,25,26]. On the other hand, strong evidence of APOBEC targeting the Ebola virus is currently not evident [27]. Similarly, no significant prevalence of APOBEC-specific mutations has been reported in influenza to date, although the expression of APOBEC proteins seems to be elevated upon influenza infection [28,29]. The dual role of ADAR and APOBEC enzymes (as players in antiviral response and as drivers of viral evolution) emphasizes the complexity of host–virus interactions.

## 3. Host–Virus Interactions

Targeting viral proteins that facilitate recognition and entry into the host cell, viral RNA replication and transcription, and virion release is a major antiviral strategy deployed by administered SARS-CoV-2, Ebola, and influenza drugs. Great effort is employed in studying the molecular mechanisms and structural determinants used throughout the viral lifecycle, as it reveals novel vulnerabilities. During their lifecycle, RNA viruses interact with a vast number of host proteins, as demonstrated by virus–host interactome studies of SARS-CoV-2, Ebola, and influenza. The dependencies on host factors might provide yet another druggable option [30,31,32]. Thus, we review the stages of RNA viral infection and interactions with the host cell factors during these stages commonly exploited by antiviral drugs, as well as those with potential to be utilized for this purpose.

### 3.1. Recognition and Entry

**Figure 2:**
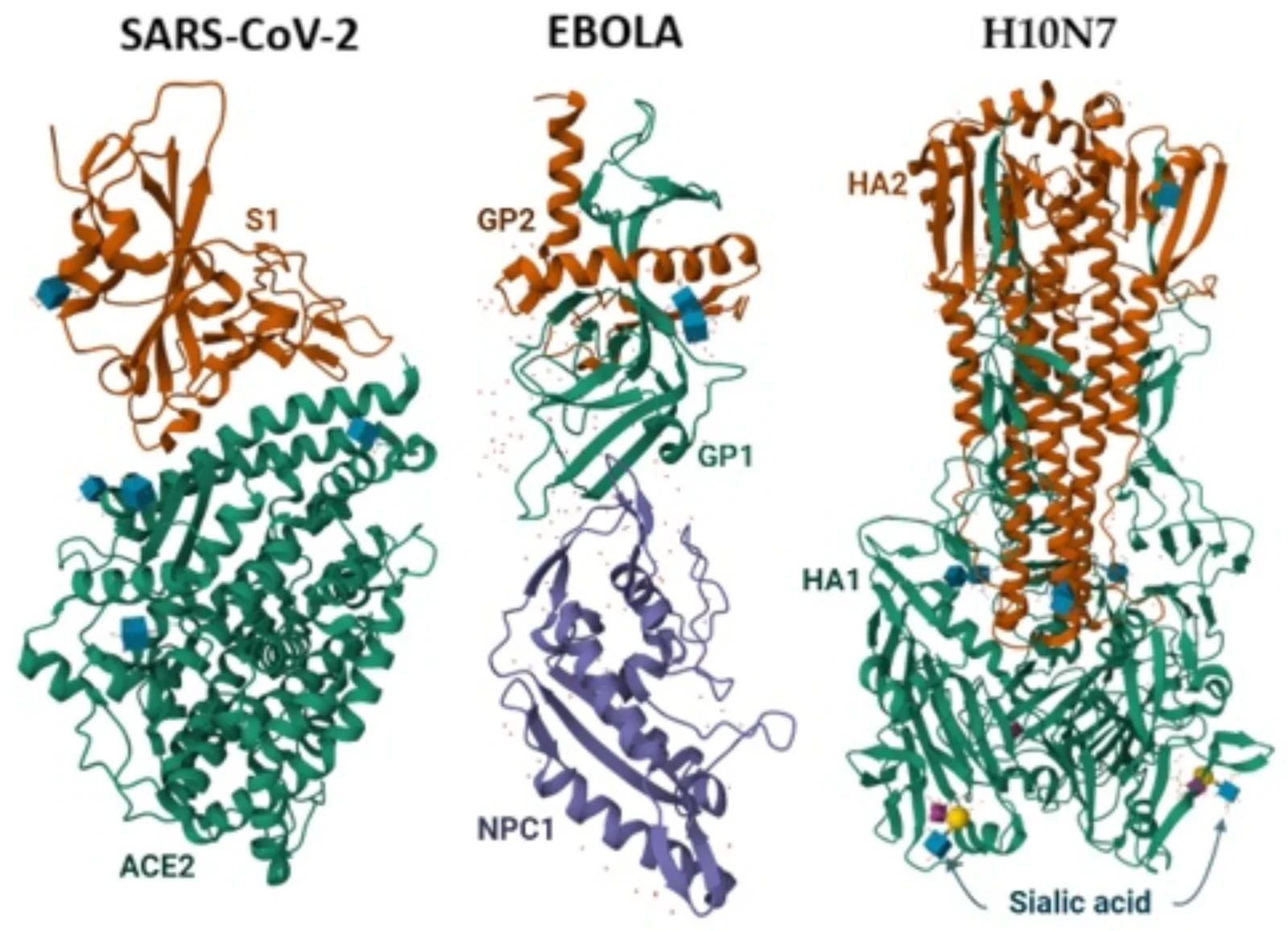
Interaction of SARS-CoV-2 (PDB ID: 6M0J), Ebola virus (PDB ID: 5F1B) and H10N7 influenza virus (PDB ID: 6TVF) glycoproteins with host cell receptors (left to right).

Host–virus interaction begins with the recognition of host cell surface proteins by viral surface proteins, which triggers entry. In the case of SARS-CoV-2, binding to the cell surface and membrane fusion is facilitated by the S glycoprotein, a homotrimer protein composed of S1 and S2 subunits [33]. While S2 serves to secure the protein within the viral envelope, the S1 subunit is the one that will attach to the host receptor, specifically angiotensin-converting enzyme 2 (ACE2), as seen in Figure 2 [34]. The receptor binding domain (RBD) of S1 is composed of two domains, the core domain (five-strand antiparallel beta sheet flanked by short alpha helices) and the receptor binding motif, which interacts with ACE2 [35]. The RBD has two conformations: “up”/“open”, in which it is accessible for receptor binding, and “down”/“closed”, in which it is “shielded”. After the interaction between RBD and ACE2 has been established, the S protein undergoes two cleavage steps by the host’s proteases that trigger extensive conformational alterations of the S protein and allow it to fuse with the host cell’s membrane. Firstly, the protein is cleaved at the boundary between the S1 and S2 subunits by the furin protein. Then, for the fusion to take place, a site within the S2, called the S2’ site, is also cleaved. Here, two possibilities exist. The S2’ site is either cleaved by transmembrane protease serine 2 (TMPRSS2), and the fusion happens at the host cell surface, or the cleavage is done by cathepsin L, and the fusion takes place at the endosome membrane (a less common mechanism). The fusion proceeds as the S2 fusion peptide gets inserted into the host membrane and refolds, pulling the membranes together, after which the RNA is released into the cytoplasm [36,37].

The attachment, entry, and release of genetic material of the Ebola virus are mediated by its sole surface protein—Ebola glycoprotein (GP). Unlike SARS-CoV-2, the interaction with the host receptor happens intracellularly, when the virus is within the endosome or lysosome. The virus attaches to the host’s cell membrane through C-type lectins, which associate with the glycosylated regions of GP, and phosphatidylserine receptors that interact with the exposed viral phosphatidylserine [38]. Following attachment, the virus may enter the host cell in different ways, mainly through a macropinocytosis-like pathway [39,40]. GP is a trimer of heterodimers composed of two subunits: a receptor-binding GP1 and the fusion mediator GP2. GP1 and GP2 are linked by a disulfide bond and non-covalent forces. The base, receptor-binding domain (RBD), glycan cap, and mucin domain comprise the GP1 subunit [41]. Similar to SARS-CoV-2, the receptor-binding domain is not readily accessible to its receptor, Niemann–Pick C1 (NPC1) [42]. After the virus is incorporated into the endosome or lysosome, cleavage by cysteine proteases removes the mucin and glycan cap, exposing the RBD [43]. Only then is RBD able to bind to its receptor, NPC1, and a conformational change is induced (Figure 2) [43]. Although the GP2 domain possesses the structural determinants needed for membrane fusion, the exact mechanism is yet to be confirmed [44]. It is suggested that the mechanism of virus–host membrane fusion might be akin to other viral fusion mechanisms [44].

The influenza entry machinery and mechanism are similar to those previously described. Its recognition, entry, and membrane fusion are mediated by influenza hemagglutinin (HA), a glycosylated trimeric protein embedded in the viral membrane [45]. Each monomer is made up of two disulfide-bonded subunits, HA1 and HA2. The HA1 subunit contains the receptor-binding domain and is located at the top of the HA2, while the HA2 subunit contains the membrane fusion polypeptide. The membrane-proximal part of the HA is characterized by a stem-like alpha-helical structure, containing residues of both HA1 and HA2. The receptor-binding domain is located in the membrane-distal globular region of HA, composed of HA1 residues and characterized by a beta-sheet structure. The receptor-binding site within the receptor-binding domain interacts with the HA receptor, sialic acid (Figure 2) [46,47]. After binding to its receptor, the influenza virus is imported into the host cell via endocytosis. Unlike SARS-CoV-2 and the Ebola virus, the influenza virus surface protein does not undergo cleavage by host proteases prior to the conformational change that enables the exposure of the fusion machinery [48]. The refolding is instead triggered by the decrease in the endosomal pH, leading to the unveiling of the fusion peptide of HA2 [48,49]. The fusion peptide is inserted into the endosomal membrane, while the rest of HA2 remains embedded in the viral envelope. The membranes are brought closer together by HA2, folding back into a hairpin structure, are fused, and the viral genome is released [50].

SARS-CoV-2, Ebola virus, and influenza virus all use class I fusion glycoproteins to mediate host cell entry. The SARS-CoV-2 spike (S) protein, Ebola virus glycoprotein (GP), and influenza virus hemagglutinin (HA) are trimeric proteins, each consisting of a receptor-binding subunit (S1, GP1, and HA1, respectively) and a membrane-fusion subunit (S2, GP2, and HA2, respectively) The SARS-CoV-2 receptor, angiotensin-converting enzyme 2 (ACE2), and influenza virus receptors, sialic acid-containing glycans, are located on the host cell surface and mediate initial attachment and entry. In contrast, the Ebola virus glycoprotein interacts with its intracellular receptor, Niemann–Pick C1 (NPC1), after endocytic uptake, whereas initial attachment to the cell surface is mediated by multiple attachment factors rather than NPC1 [51,52,53,54]. The host receptor interaction of SARS-CoV-2, Ebola virus, and influenza virus is mediated by class I fusion glycoproteins [55]. Because of this, some commonalities in the overall molecular mechanism, as well as the structure of their respective glycoproteins, are present. SARS-CoV-2 S protein, Ebola glycoprotein, and influenza hemagglutinin are trimeric proteins with a subunit allocated for receptor binding and a subunit for membrane fusion. The receptor binding is followed by a structural remodeling, which reveals the fusion machinery. While for influenza HA, it is triggered by a change in endosomal pH, the remodeling of the SARS-CoV-2 S protein and the Ebola GP depends on cleavage by host proteases [35,42,47]. The S protein activation mechanism is more complex than that of influenza and Ebola virus, requiring first the cleavage at the interface between the two subunits, and then within the fusion subunit. The viral entry mechanisms also differ in the attachment factor. For SARS-CoV-2 and the influenza virus, the attachment to the host cell is primarily mediated by glycoprotein binding to specific receptors on the host cell membrane, ACE2 and sialic acid, respectively [35,47]. The Ebola virus, however, attaches to the host cell membrane through interaction with lectins and phosphatidylserine receptors, and binds its receptor NPC1 after it has been internalized [42]. The mechanism of membrane fusion is facilitated by similar fusion machinery and, therefore, shared by these viruses, but the site may differ. SARS-CoV-2 fuses at the host cell membrane but has also been shown to be able to fuse at the endosomes; Ebola virus fuses in the late endosomes or lysosomes, and influenza viruses at the acidified endosomes [37,44]. In addition, further host interactions during recognition and entry of SARS-CoV-2, Ebola, and influenza are presented in Table 1.

### 3.2. Replication and Transcription

After the viral particles of SARS-CoV-2 have entered the host cell cytoplasm, their genomes are dissociated from the nucleocapsid (N) protein. As a positive-sense RNA virus, SARS-CoV-2 RNA is immediately used as a template for translation by the host cell’s ribosomes. Two 5’ viral genomic sequences, ORF1a and ORF1ab, are used to construct the polyproteins pp1a and pp1ab, respectively [56]. The cleavage of the polyproteins by SARS-CoV-2 protease proteins (such as the main protease or MPRO) yields non-structural proteins (nsps), which form the RTC, as previously discussed [57]. The RTC synthesizes new RNA, either continuously to form new genomes to be packaged in virions or discontinuously to transcribe the mRNA needed to form structural and accessory proteins [58]. The viral RNA is not synthesized freely in the cytoplasm. Instead, SARS-CoV-2 takes advantage of the host cell’s endoplasmic reticulum membranes to form dense replication centers termed double-membrane vesicles (DMVs), formed primarily by nsp3 and nsp4 [59]. A special feature of this virus is its proofreading ability, mediated by nsp14 and its cofactor nsp10. The nsp14 proofreading works by exonucleolytic removal of erroneous nucleotides at the 3’ end of nascent RNA, ensuring the stability of the unusually large genome of SARS-CoV-2 [60]. The newly synthesized RNA is then modified by the addition of a 5’ cap, which mimics host RNA, thereby enabling translation by host ribosomes and avoiding immune sensors. The capping is mediated by nsps, including the NiRAN domain of nsp12, nsp13, nsp14, and nsp16 [10,61,62,63,64]. The nascent RNA is polyadenylated to increase stability and translation by host machinery, but the exact mechanism of this modification in SARS-CoV-2 is not known [56]. Furthermore, it is suggested that SARS-CoV-2 exploits the poly(A) binding proteins (PABPs), the host’s translation factors, in addition to nsp1 inhibiting host mRNA translation and promoting mRNA degradation [64,65].

Unlike SARS-CoV-2, the Ebola virus genome is a negative-sense RNA and cannot immediately be translated by host ribosomes. Furthermore, the Ebola genome is not free; instead, it is encapsidated by nucleoprotein (NP) to form a ribonucleoprotein (RNP) [66]. The L protein and VP35 cofactor form the main replication/transcription machinery, while VP30 is an important regulator [67]. It is suggested that in its phosphorylated state, it binds VP35 and the L protein to facilitate the transcription state, while without it, the replication state is promoted [68,69]. After entry, transcription occurs first to convert the negative-sense RNA genome into positive-sense viral mRNAs by RdRp, after which the host ribosomes are used to make viral proteins. Like SARS-CoV-2 mRNA, the Ebola virus mRNAs are capped and polyadenylated [66]. From the positive-sense antigenome, the Ebola genome is produced. RNA synthesis mainly occurs in the viral inclusion bodies, which are described in more detail below [70]. Once replicated, the genome is encapsidated by NP to form a mature nucleocapsid. The nucleocapsids are then transported out of the inclusion bodies to the plasma membrane, where they are packaged and released from the host cell by budding off. The transport from the inclusion bodies, packaging, and release are supported by VP40, VP35, and VP24 [71,72,73]. Additionally, the host endosome recycling pathway is leveraged by the Ebola virus for viral particle transport to the membrane and subsequent release through interaction of VP40 with Rab11, a host cell GTPase [74].

Like the Ebola virus, influenza has a negative-sense RNA genome packaged as a nucleoprotein. However, the influenza virus genome is partitioned and requires a mechanism of assembly [75]. After viral entry and release into the host cytoplasm, the RNPs are transported into the host cell nucleus, where influenza RNA synthesis takes place. Interestingly, the influenza virus requires the host ANP32 proteins for RdRp complex assembly, RNA synthesis, and encapsidation [76]. The influenza virus utilizes a distinct transcription mechanism termed “cap snatching”, in which the host mRNA is used as the transcription primer. The process starts with PA cleaving nascent mRNA from the host’s DNA-dependent RNA polymerase II and transferring it to PB2. RNA is then synthesized by PB1 and finally polyadenylated [13,77]. Next, the newly synthesized viral RNA is exported to the cytosol, where it is translated by host ribosomes. The positive antigenome and new copies of the genome are synthesized by PB1, and then coated by NP [13]. As the virus contains 8 RNA genome segments, it has to supply each virion with a copy of each one. The RNA-RNA interactions between the segments support the selection and packaging, and signal sequences in different RNA partitions are required for correct assortment [78,79]. An important platform that facilitates influenza genome assembly through ribonucleoprotein interactions is Rab11 biomolecular condensates [80,81]. As ribonucleoproteins leave the nucleus (mediated by viral proteins), they associate with Rab11, exploiting the host endosome recycling mechanism for transport to the plasma membrane, where they bud off [82,83,84]. As the virions bud off, they remain attached to the host cell’s sialic acid and are released through cleavage by the viral surface neuraminidase enzyme (NA) [85]. Additional examples od virus–host interactions during viral replication, transcription, assembly, and release are presented in Table 2.

Structures such as those formed by RNP and Rab11, Ebola viral inclusion bodies, and SARS-CoV-2 replication/transcription centers are examples of biomolecular condensates, an important newly emerging concept in host–virus interactions and novel antiviral therapeutic development [86]. Biomolecular condensates are phase-separated compartments, dense with certain proteins and nucleic acids. They are also termed membraneless organelles because they serve as sites for specific interactions [86]. A major process by which biomolecular condensates form is liquid–liquid phase separation, where droplets dense with solute partition out of the surrounding solvent [87]. Intrinsically disordered proteins (IDPs) are characterized by not having a single stable structure and are often found in viruses, aiding in taking over the host cellular machinery [88]. IDPs are frequently found in phase-separated structures, and their multivalency is identified as a key driver [89]. Viral biomolecular condensates observed in SARS-CoV-2, Ebola, and influenza-infected cells and related to their replication, transcription, and egress are reviewed here. Furthermore, the interactions of these viruses with the host’s biomolecular condensates, such as stress granules, modulating the immune response, are discussed in the next section.

The SARS-CoV-2 nucleocapsid (N) protein has intrinsically disordered regions and has been demonstrated to undergo phase separation [90]. It is suggested that the formed molecular biocondensates have important roles in efficient viral packaging and replication/transcription center formation [90]. A similar phenomenon is present in Ebola virus inclusion bodies, biomolecular condensates formed through LLPS driven by Ebola virus nucleoprotein (NP), a nucleocapsid protein with IDRs [91]. These inclusion bodies have been demonstrated to be important for RNA synthesis, especially as replication sites for the Ebola virus, although transcription takes place outside of the inclusion bodies as well [70]. Alongside Ebola proteins (NP, VP35, VP30, and L protein) and RNA, it was demonstrated that the host’s CAD protein complex, involved in de novo synthesis of pyrimidines, is recruited to the inclusion bodies, suggesting a high demand for RNA building blocks inside them, consistent with the biomolecular condensates being major sites of RNA synthesis [92]. The intrinsically disordered regions of the SARS-CoV-2 nucleocapsid (N) protein that drive biomolecular condensate formation have been identified, including the N-terminal intrinsically disordered region (NTD), the central linker intrinsically disordered region (LKR), and the C-terminal intrinsically disordered tail (CTT), which together regulate phase separation and RNA binding, but for Ebola inclusion bodies, NP oligomerization was identified as a key driver rather than its IDRs [91,93]. Ebola’s VP35 protein, which contains intrinsically disordered regions, forms a complex with NP and phase separates to form inclusion bodies and is an important modulator of NP-RNA binding and Ebola virus RNA synthesis [94,95,96]. The same protein has also been shown to hijack the host AKIP1-PKA-CREB1 pathway, with CREB1 being recruited to the inclusion bodies where it supports viral replication [97]. Moreover, the Ebola virus utilizes host proteins for mRNA export from the inclusion bodies through NP-nuclear RNA export factor 1 (NXF1) interaction [98]. Both of these host–virus interactions, as well as the interaction with the CAD complex, have been suggested to be promising therapeutic avenues [92,97,98]. Compared to the Ebola virus and SARS-CoV-2, the biomolecular condensates and the related interactions of influenza are suggested to be related to genome packaging rather than RNA synthesis support [84]. The less extensive research on influenza biomolecular condensates could be partly due to the technical challenges, and novel methods of studying these dynamic structures could contribute to novel antiviral strategies [99].

### 3.3. Immune Evasion

Throughout their lifecycle, viruses deploy a variety of molecular mechanisms to evade the host’s defenses. While distinct in their execution, SARS-CoV-2, influenza, and Ebola virus share certain targets, including host pathways and proteins, of immune evasion strategies. The viral components are recognized by innate immune system factors, such as retinoic acid-inducible gene I (RIG-I) and melanoma differentiation-associated gene 5 (MDA-5), which induce signaling cascades, culminating in interferon production [100]. Interferons (IFNs) then activate the JAK-STAT pathway, resulting in activation of interferon-stimulated genes (ISGs) and creation of an antiviral state [101]. SARS-CoV-2, influenza, and Ebola virus target different players within different stages of this cascade in order to evade or decrease the immune response and ensure their replication. Understanding the interactions of viruses with immune system factors reveals antiviral strategies.

Evasion of viral RNA sensing in the host cytosol by Ebola virus is mediated mainly by VP35. Its interferon antagonism consists of viral RNA masking and interferon signaling disruption through phosphorylation impairment. Viral RNA masking is mediated by the VP35 IFN inhibitory domain (IID), preventing recognition by RIG-I/MDA-5 and weakening the IFN responses [102]. Additionally, the phosphorylation of transcription factors IRF3 and IRF7 that drive interferon-1 expression is blocked by VP35 mimicking kinase substrate (IRF3) or promoting SUMOylation by exploiting the host SUMOylation machinery (IRF7), thereby suppressing interferon transcription [103,104,105]. While the Ebola virus primarily hides its RNA to evade immune sensing, the influenza virus non-structural protein 1 (NS1) disrupts the ubiquitination needed for RIG-I signaling and subsequent IFN production [105]. NS1 blocks the assembly of TRIM25 (Tripartite Motif-Containing Protein 25), the enzyme mediating RIG-I ubiquitination, preventing efficient RIG-I ubiquitination and downstream IFN induction [106]. On the other hand, SARS-CoV-2 utilizes a multifaceted strategy of immune-sensing evasion, with many protein players, including ORF9b, Nsp15, M protein, and other structural and nonstructural proteins, and multiple pathways, including RIG-I (Retinoic acid-inducible gene I), MDA5 (Melanoma differentiation-associated protein 5), MAVS (Mitochondrial Antiviral Signaling Protein), and TBK1 (TANK-binding kinase 1), which are targeted [107,108]. Similar to the Ebola virus, one of the immune-sensing evasion strategies of SARS-CoV-2 is inhibition of IRF3 phosphorylation; however, it does so by interfering with the upstream signaling, rather than as a substrate decoy [109]. Thus, although all three viruses target the same immune mechanisms, they do so utilizing distinct molecular strategies.

After interferon is produced, the viruses evade the host immune response by affecting the cellular response to interferons, mainly by modifying the JAK-STAT pathway and reducing the transcription of interferon-stimulated genes (ISGs). Ebola VP24 disrupts the pathway by binding karyopherin-α proteins, host nuclear transport proteins, impairing their ability to import STAT1 and limiting the ISG transcription [110,111]. A similar outcome achieved through a different molecular mechanism is observed with SARS-CoV-2 ORF6, which interferes with the STAT1/STAT2 nuclear import by exploiting host Nup98-Rae1 complexes at the nuclear pore [112,113]. The influenza virus, on the other hand, targets the signaling in the JAK-STAT pathway rather than nuclear import. The PB2 subunit of the influenza virus RdRp promotes ubiquitination and proteasomal degradation of JAK1, reducing the activation of STAT1/STAT2 and ISG production [114].

Another viral strategy deployed to escape the host’s immunity is suppressing the host’s mRNA or protein production, while maintaining viral gene expression. For example, the SARS-CoV-2 nsp1 C-terminal domain binds the mRNA channel of the 40S ribosome subunit, where it blocks mRNA binding. This results in decreased host translation and production of antiviral proteins [65,115]. The influenza virus performs host gene expression shutoff by multiple molecular mechanisms. Firstly, a portion of the nascent host mRNA is eliminated during the cap-snatching stage of influenza replication and transcription [13]. In addition to this, the NS1 protein prevents the processing and export of nascent host mRNA. The NS1 protein binds CPSF30 (Cleavage and Polyadenylation Specificity Factor 30) protein, a part of the host pre-mRNA processing apparatus, leading to inhibition of host pre-mRNA cleavage and polyadenylation, including interferon pre-mRNA [116]. Furthermore, PA-X protein suppresses host protein synthesis in the cytoplasm. PA-X is a protein made from the same gene as the PA subunit of influenza RdRp through ribosome frameshifting. While it lacks the C-terminal domain found in the RdRp counterpart, it possesses the endonuclease domain. Unlike SARS-CoV-2 nsp1, which inhibits protein translation, the suppression of host protein synthesis by influenza PA-X is achieved by selective endonucleolytic degradation of host mRNA [117,118]. The Ebola virus has not been demonstrated to utilize specific host gene-expression shutoff factors, like influenza and SARS-CoV-2.

**Figure 3:**
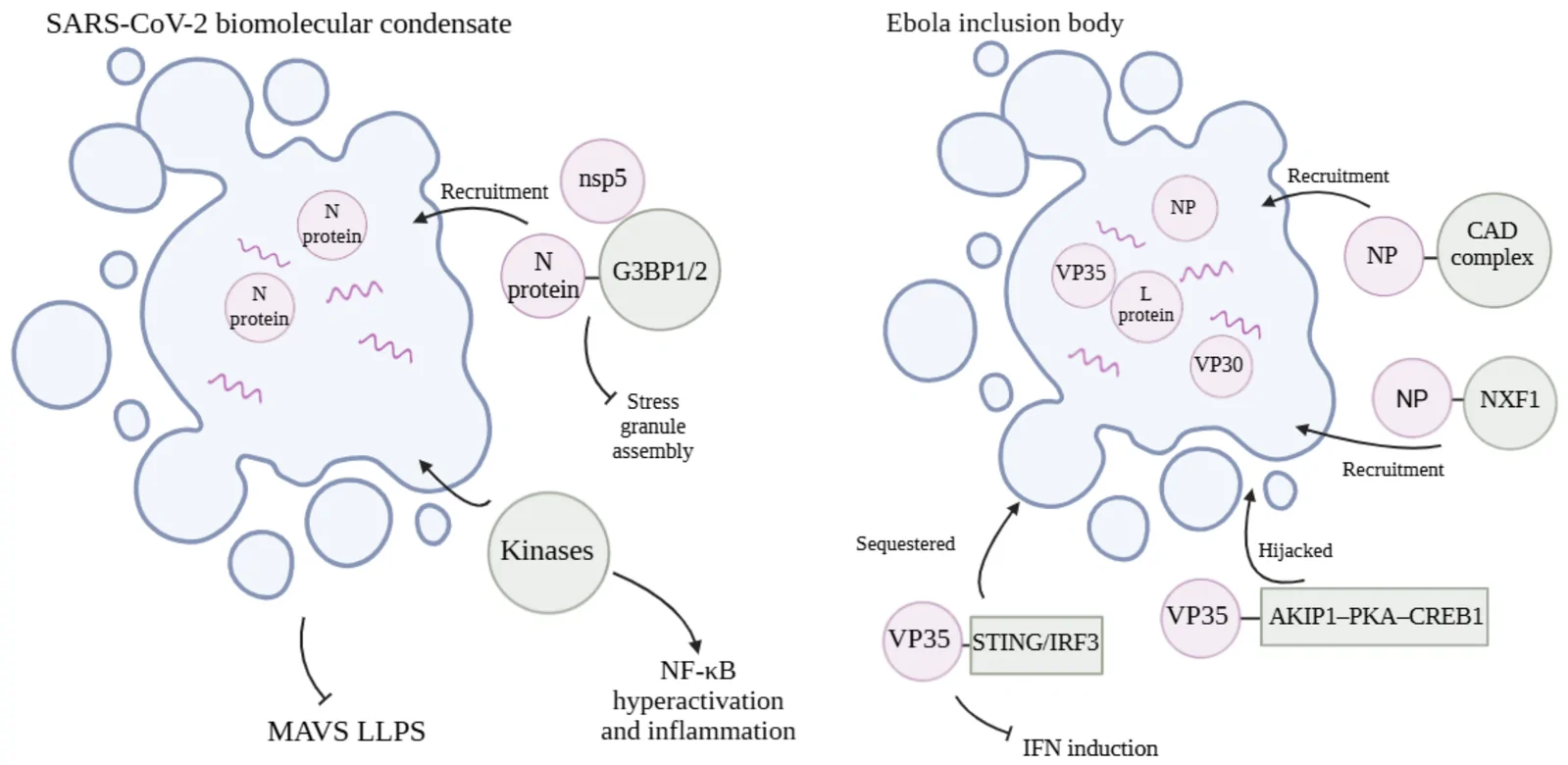
Summary of virus–host interactions related to SARS-CoV-2 and Ebola virus LLPS compartments. SARS-CoV-2 and Ebola virus use biomolecular condensates as sites of RNA synthesis and for host immune evasion. Host factors are recruited by viral proteins into the condensates. Influenza biomolecular condensate host interactions are currently not clearly mapped and are therefore not represented. Created with BioRender.com.

Biomolecular condensates provide yet another mechanism of immune evasion to these viruses. Upon cellular stress or infection, the cells form biomolecular condensates of protein and RNA known as stress granules. The stress granules can recruit immune sensors and signaling factors, such as RIG-I, MDA5, PKR, MAVS-associated signaling components, and G3BP proteins [101]. SARS-CoV-2, Ebola, and influenza viruses evade the host immune system by directly disrupting stress granules or by viral condensates interfering with the host. Direct disruption of host stress granules is caused by viral proteins interacting with core stress granule proteins in cases of SARS-CoV-2 and Ebola, and by distinct molecular mechanisms in influenza. SARS-CoV-2 N protein and nsp5 suppress the formation of host stress granules through G3BP1 and G3BP2 interactions, and Ebola VP35 through interactions with G3BP1, eIF3, and eEF2, thereby weakening the antiviral response [119,120,121]. Stress granule formation inhibition by influenza is mediated by NS1, NP, and PA-X [122]. Furthermore, the formation of SARS-CoV-2 biomolecular condensates interferes with MAVS LLPS, whereas Ebola VP35 secludes IRF3 into its inclusion bodies, both reducing interferon signaling [123,124]. SARS-CoV-2 condensates are also utilized to hijack host signaling kinases, leading to NF-κB hyperactivation and inflammation [125]. The role of stress granules in mediating antiviral responses has been elusive and complicated. Whereas early reports propose that these membrane-less structures facilitate anti-viral immune responses, a subsequent study suggests that stress granules act to prevent excessive innate immune activation during viral infections, and they may regulate viral replication via mechanisms independently of the innate immune pathway [126]. The summary of virus–host interactions during innate immune evasion can be observed in Table 3 and Figure 3.

## 4. Antiviral Strategies

Most viral pathogens represent a serious health risk for humans. Non-pharmacological measures like increased hygiene, social distancing, and facial protection are not enough to avoid infection. Successful eradication of viruses is possible with vaccines or antiviral drugs, which reduce the morbidity and mortality caused by a viral infection [7]. Different antiviral strategies are employed against different viruses, and many structural and mechanistic determinants, as well as the host–virus interactions, are explored as potential antiviral targets. Here, the most important antiviral strategies for Ebola, SARS-CoV-2, and influenza are reviewed.

As the first step of infection, viral entry into host cells is an important target of antiviral drugs because it stops further viral multiplication and decreases the chance of downstream resistance. For the Ebola virus, the antiviral strategy involves targeting the virus’s entry through neutralization of its sole surface protein, GP, with monoclonal antibodies [127]. Current studies suggest that antibodies specific to GP are among the most effective treatments [128]. Notably, present mAb (monoclonal antibody)-based treatments designed as glycoprotein inhibitors encompass Inmazeb™ (REGN-EB3) and Ebanga™ (mAb114), approved in 2020 [129]. Earlier, ZMapp, an antibody cocktail comprising three distinct monoclonal antibodies that selects the virion’s exterior glycoprotein, impeding the development of Ebola Virus Disease, was used. A randomized controlled trial in 2019, however, demonstrated the superiority of mAb114 and REGN-EB3 to ZMapp for mortality reduction in Ebola virus disease [128]. Ansuvimab-zykl (ansuvimab), previously known as mAb114, was created by the Vaccine Research Center and aided by the US National Institutes of Health for therapy management [130]. The drug works by binding the epitope within the receptor-binding site in the GP1 subunit, preventing its crucial interaction with the NPC1 receptor [130,131]. REGN-EB3, the inaugural drug that was approved by the FDA for adults and kids, is composed of three fully human monoclonal antibodies that hinder the virus’s adherence to the proteins in the host. Targeting a single epitope allows viruses to develop resistance by mutation. This antibody cocktail, consisting of three antibodies, each one targeting a nonoverlapping epitope, slows down the development of escape mutants [132]. Neutralizing monoclonal antibodies and antibody cocktails that target the SARS-CoV-2 S glycoprotein and prevent its binding to ACE2 exist and have been approved for use by the FDA. However, due to rapid mutation, resistance to all of them led to their withdrawal [133]. Similarly, due to the prevalence of escape mutants and the high variability of influenza HA, the receptor binding has not been a successful target for influenza treatment. However, the inhibition of fusion mediated by HA may be a promising antiviral entry target [134,135]. A potential alternative to monoclonal antibodies, which are less sensitive to mutations, is small-molecule inhibitors [133]. Although many small molecules are tested against the entry pathways of Ebola, SARS-CoV-2, and influenza, none are currently in clinical use [136,137].

Targeting RNA-dependent RNA polymerases is another important antiviral strategy, as this enzyme is indispensable for replication and transcription of RNA viruses and structurally conserved with palm, fingers, and thumb domains [138]. While some antiviral strategies targeting RNA synthesis have broad activity, others target specific mechanisms/structures of a particular virus (for example, baloxavir, as discussed below and presented in Table 4). Remdesivir is an adenosine analog prodrug initially created for the treatment of the Ebola virus (EBOV). It was shown to reduce the replication of human and animal coronaviruses in vitro and in preclinical studies. After entering the cells, remdesivir is converted to remdesivir triphosphate (RTP) and incorporated as a nucleotide triphosphate (NTP) into a growing RNA chain. The RNA polymerase elongates the chain for a few more nucleotides before the RNA synthesis is stalled [139,140]. Although originally investigated for its use in EBOV, remdesivir is currently approved for SARS-CoV-2 treatment, whereas mAb114/Ebanga and REGN-EB3/Inmazeb are recommended for EBOV, as previously discussed. Molnupiravir (Lagevrio), created in collaboration with Merck and Ridgeback Biotherapeutics, is another RdRp inhibitor used against SARS-CoV-2. Molnupiravir, like remdesivir, is a nucleoside analogue, although they function in quite distinct ways. Remdesivir serves as a “chain terminator” by interfering with RNA chain elongation, while molnupiravir functions as a mutagenizing agent, causing an “error catastrophe” throughout viral replication and so limiting the production of contagious viral fragments [141]. Molnupiravir is studied as an antiviral strategy against other viruses, including the Ebola virus and influenza, but is not approved for clinical use against these viruses [142]. Recently approved drugs targeting the influenza virus are baloxavir marboxil and favipiravir. Baloxavir is converted to baloxavir acid by host enzymes upon entry, and baloxavir acid binds the PA endonuclease active site, interfering with its “cap-snatching” mechanism [143]. Pimodivir is another drug targeting influenza’s “cap-snatching” mechanism. It does so by binding PB2 instead of PA. Pimodivir, however, was demonstrated not to have significant clinical benefit, and baloxavir remains the recommended influenza treatment [144]. Because baloxavir and pimodivir target a distinct molecular mechanism present in influenza, their utility for SARS-CoV-2 and Ebola is limited. Favipiravir is a nucleoside analog that undergoes intracellular phosphoribosylation to favipiravir-RTP (favipiravir-ribofuranosyl-5′-triphosphate), its active form, after oral ingestion. Its mechanism of action is similar to that of molnupiravir, getting incorporated into the RNA and subsequently causing errors to be made during RNA synthesis. Favipiravir is approved for clinical use for exceptional cases not responding to standard Ebola treatment in Japan and was investigated for use against SARS-CoV-2 and influenza, although significant efficacy against these two viruses was not found [145,146,147]. A distinct molecular mechanism of SARS-CoV-2 disrupted as part of an antiviral strategy is its processing of polyproteins. Nirmatrelvir is an oral medication that acts as an inhibitor of the SARS-CoV-2 main protease protein, disrupting polyprotein cleavage. Although proven to be clinically useful, there has been a concern about the development of resistance by SARS-CoV-2, as was the case with other antivirals, and multiple resistance mechanisms were recorded in vitro [148,149]. In addition to baloxavir, oseltamivir, zanamivir, and peramivir are routinely used influenza antivirals. These belong to the same class of compounds called influenza neuraminidase inhibitors. As neuraminidase inhibitors, oseltamivir, zanamivir, and peramivir bind the neuraminidase active site, blocking the cleavage and impairing the release of the influenza virions from the host cell sialic acid, preventing further viral spread [150].

The development of novel antiviral strategies for controlling infections in humans is crucial. RNA viruses, such as Ebola, influenza, and SARS-CoV-2, evolve rapidly, making antiviral resistance a significant challenge in infection treatment. Targeting host structures important for the viral lifecycle may be important for limiting the resistance to antiviral drugs seen with many previously effective antivirals, as well as increasing the spectrum of activity to multiple viral strains [151]. For example, utilizing a TMPRSS2 inhibitor to block the SARS-CoV-2 S protein cleavage needed for conformational change induction and membrane fusion might be a novel antiviral strategy [152]. Similarly, targeting host factors involved in the influenza lifecycle, such as the ANP32 proteins necessary for its replication or transport pathways, is a potential avenue. Still, research into the safety, specificity, and effectiveness of host-directed antiviral strategies is needed before clinical deployment [153]. Host–virus interactomes here provide insights into potentially useful antiviral structures and molecular pathways for SARS-CoV-2, Ebola, and influenza [30,31,154,155]. Furthermore, the host targets for which clinically approved drugs already exist can be uncovered using host–virus interaction maps [156]. This approach is especially useful when timely drug candidates are needed, as was the case with the COVID-19 pandemic [153].

CRISPR, a nucleic acid-targeting technique, can also be effectively applied as an antiviral strategy to eliminate RNA viruses. It is an associated protein 13 (Cas13) system that has two functional parts—single-guide RNA (sgRNA) and the endonuclease Cas13 that is directed to a particular region within the RNA molecule. It is important to mention that there is a risk of the development of off-target effects with this technique. Despite its promise, several challenges remain for clinical translation, including efficient and tissue-specific delivery of the CRISPR-Cas13 machinery, minimizing off-target RNA cleavage, and ensuring long-term safety without eliciting undesirable immune responses. Likewise, therapeutic strategies targeting biomolecular condensates must achieve high specificity to selectively disrupt virus-associated condensates while preserving physiological host condensates that are essential for normal cellular functions [157]. Overcoming this and other challenges related to CRISPR editing technology will be necessary before its clinical deployment against RNA viruses, but it is a promising novel strategy [158,159]. Another novel antiviral strategy explored for eliminating RNA viruses is targeting the biomolecular condensates or LLPS they form, such as the replication condensates and hijacked stress granules. Disrupting these compartments can limit the replication of RNA viruses, including SARS-CoV-2, Ebola, and influenza, and rescue innate immune system signaling [90,160]. Instead of distinct structural targets in traditional antiviral drugs, the potency of proteins driving LLPS and biomolecular condensate formation or the material properties of condensates is altered in this approach [160].

Despite encouraging preclinical advances, most emerging antiviral strategies remain at an early stage of clinical development. Host-directed therapies, CRISPR-Cas13-based antivirals, and biomolecular condensate-targeting approaches have demonstrated promising antiviral activity in cell culture and animal models, but none have yet received regulatory approval for the treatment of RNA virus infections. Their clinical translation is challenged by efficient and tissue-specific delivery, target specificity, potential off-target effects, safety concerns related to modulation of essential host pathways, and the rapid emergence of viral escape variants. Continued optimization of delivery systems improved molecular selectivity, and rigorous evaluation in clinical trials will be required before these approaches can become viable therapeutic options [161,162]. Several emerging strategies are presented in Table 5.

## 5. Conclusions

The ability of RNA viruses to rapidly evolve allows easier evasion of the immune system and antiviral resistance, making them one of the most significant threats to public health. In addition, zoonotic potential contributes to the persistent threat of pathogenic RNA virus outbreaks and pandemics. Licensed antiviral drugs against the diseases caused by pathogenic RNA viruses, such as SARS-CoV-2, Ebola, and influenza viruses, are available. Current approaches target viral structures to disable crucial molecular mechanisms, such as RNA synthesis, host cell entry and fusion, processing, and virion release. However, rapid mutation through RNA-dependent RNA polymerase, host-mediated editing mechanisms, and viral recombination challenge effective treatment, leading to therapeutic failure. Some of the emerging antiviral strategies, such as host-directed targeting, LLPS disruption and modification, and CRISPR-Cas13 RNA targeting, show promise in addressing the current treatment limitations, but the feasibility of clinical utilization of these novel antiviral strategies is yet to be determined. These approaches need further optimization to address safety concerns, delivery efficiency, off-target effects, and long-term clinical outcomes. Continued research into viral evolution, structural and molecular determinants, and host–virus interactions is essential for improving our ability to prevent and treat diseases caused by highly pathogenic RNA viruses.

## Figures and Tables

**Table 1:** Summary of virus–host interactions during recognition and entry of SARS-CoV-2, Ebola, and influenza.

Virus	Host Factor	Viral Factor	Interaction/Role
**SARS-CoV-2**	ACE2	Spike S1 RBD	Receptor binding
	Furin	Spike S1/S2 site	Spike priming cleavage
	TMPRSS2	Spike S2′ site	Fusion-activating cleavage at the cell surface
	Cathepsin L	Spike S2′ site	Fusion-activating cleavage in the endosome
**Ebola virus**	C-type lectins	GP glycosylated regions	Cell attachment
	Phosphatidylserine receptors	Viral phosphatidylserine/virion surface	Cell attachment
	Cathepsins/cysteine proteases	GP1 glycan cap and mucin domain	Proteolytic cleavage exposing the RBD
	NPC1	Cleaved GP1 RBD	Intracellular receptor binding
**Influenza A virus**	Sialic acid	HA1 receptor-binding site	Receptor binding
	Endosomal low pH	HA2	Fusion peptide exposure and membrane fusion

**Table 2:** Summary of virus–host interactions during viral replication, transcription, assembly, and release.

Virus	Host Factor	Viral Factor	Interaction/Role
**SARS-CoV-2**	ER membranes	nsp3, nsp4	DMV formation
	Host translation machinery	Capped viral mRNA	Viral protein translation
	PABPs	Viral RNA/viral replication machinery	Suggested support for RNA stability and translation
	Host mRNA/translation machinery	nsp1	Host mRNA translation inhibition and degradation
**Ebola virus**	Host ribosomes	Viral mRNAs	Viral protein translation
	Rab11	VP40	Virion transport and budding support
	CAD (Carbamoyl-phosphate synthetase 2, Aspartate transcarbamylase, and Dihydroorotase) protein complex	NP, VP35, VP30, L	Recruitment to inclusion bodies for nucleotide supply
	CREB1/AKIP1-PKA-CREB1 (cAMP Response Element-Binding Protein 1/A-Kinase Interacting Protein 1, Protein Kinase A, cAMP Response Element-Binding Protein 1) pathway	VP35/inclusion body proteins	CREB1 recruitment supporting replication
	NXF1	NP	Viral mRNA export from inclusion bodies
**Influenza A** **virus**	RNA polymerase II nascent mRNA	PA	Cap-snatching cleavage
	Host-capped RNA fragment	PB2	Cap binding during cap snatching
	ANP32 proteins	Polymerase complex	Support of polymerase activity
	Rab11	Viral RNPs	RNP trafficking and genome assembly condensates
	Sialic acid	NA	Cleavage of sialic acid during virion release

**Table 3:** Summary of virus–host interactions during innate immune evasion.

Virus	Host Factor	Viral Factor	Interaction/Role
**SARS-CoV-2**	Host immune RNA sensors	5′-capped viral RNA	Immune-sensor avoidance
	Host mRNA/ribosome entry channel	nsp1	Host gene-expression shutoff
	G3BP/stress granule proteins	N protein	Stress granule modulation/condensate interaction
**Ebola virus**	TBK1/IKKε pathway	VP35	Interference with IFN induction
	IRF3	VP35	Blocked IRF3 activation/signaling
	IRF7/UBC9/PIAS1	VP35	IRF7 sumoylation modulation
	G3BP/stress granule proteins	VP35	Stress granule antagonism
**Influenza A virus**	TRIM25	NS1	Disruption of RIG-I ubiquitination
	RIG-I pathway	NS1	Suppression of antiviral sensing
	Host mRNA/gene-expression machinery	PA-X	Host mRNA degradation and translation suppression

**Table 4:** Approved antiviral strategies against SARS-CoV-2, influenza, and Ebola virus.

Virus	Lifecycle Stage	Drug	Target	Molecular Outcome
**SARS-CoV-2**	RNA synthesis/genome replication	Remdesivir	RdRp	RNA synthesis stalled
	RNA synthesis/genome replication	Molnupiravir	RdRp	Viral mutagenesis
	Proteolytic processing	Nirmatrelvir	Main protease	Viral polyproteins not properly cleaved
**Ebola virus**	Entry/fusion	REGN-EB3/Inmazeb	Ebola GP, multiple non-overlapping epitopes	GP-mediated entry neutralized; immune clearance enhanced
	Entry/receptor engagement	mAb114/ansuvimab/Ebanga	GP1 receptor-binding region	Cleaved GP cannot efficiently engage NPC1, blocking entry/fusion
**Influenza virus**	Viral transcription/mRNA synthesis	Baloxavir	PA endonuclease site	Cap-snatching blocked, reduced viral mRNA synthesis
	Virion release	Oseltamivir, zanamivir, peramivir	Neuraminidase	Sialic acid cleavage inhibited, reduced release/spread of virions

**Table 5:** Summary of the clinical status of the mentioned emerging therapies.

Emerging Strategy	Representative Target	Current Clinical Status	Main Translational Challenges
Host-directed therapies	TMPRSS2, ANP32 proteins, host trafficking pathways	Preclinical; some host-targeting compounds repurposed or evaluated in early clinical studies	Host toxicity, preservation of physiological functions, specificity, patient variability
CRISPR-Cas13 antiviral therapy	Viral RNA	Preclinical proof-of-concept	Efficient in vivo delivery, tissue targeting, off-target RNA cleavage, immunogenicity, regulatory challenges
Biomolecular condensate/LLPS targeting	Viral replication condensates, stress granules	Preclinical	Selective disruption of viral condensates without affecting physiological condensates, limited availability of specific small molecules, safety
Host interactome-guided drug repurposing	Host proteins interacting with viral proteins	Clinical evaluation for selected repurposed drugs; strategy under active investigation	Validation of targets, heterogeneous patient responses, limited antiviral efficacy of individual repurposed agents
Small-molecule viral entry inhibitors	Viral fusion/entry machinery	Preclinical to early clinical evaluation depending on virus	Viral escape mutations, optimization of potency and bioavailability

## Data Availability

No new data were created or analyzed in this study. Data sharing is not applicable to this article.
